# Spatial Distribution and Host‐Specific Patterns of Avian Haemosporidian Infections in a Semi‐Arid Region of Northeastern Iran

**DOI:** 10.1002/vms3.71047

**Published:** 2026-06-23

**Authors:** Seyed‐Reza Mirbadie, Rasoul Alimi, Eissa Soleymani, Naghmeh Dastan, Abdolmajid Gholizadeh, Amir‐Hossein Maghsood, Mohammad‐Ali Mohaghegh

**Affiliations:** ^1^ Student Research Committee Hamadan University of Medical Sciences Hamadan Iran; ^2^ School of Medicine Shahroud University of Medical Sciences Shahroud Iran; ^3^ Department of Epidemiology and Biostatistics, School of Health Torbat Heydariyeh University of Medical Sciences Torbat Heydariyeh Iran; ^4^ Department of Clinical Laboratory Science Babol Branch, Islamic Azad University Babol Iran; ^5^ Department of Parasitology and Mycology, Faculty of Medicine Mazandaran University of Medical Sciences Sari Iran; ^6^ Department of Medical Parasitology and Mycology, School of Medicine Hamadan University of Medical Sciences Hamadan Iran; ^7^ Department of Environmental Health Engineering, School of Health Torbat Heydariyeh University of Medical Sciences Torbat Heydariyeh Iran; ^8^ Department of Laboratory Sciences, School of Paramedical Sciences Torbat Heydariyeh University of Medical Sciences Torbat Heydariyeh Iran; ^9^ Public Health Research Center Torbat Heydariyeh University of Medical Sciences Torbat Heydariyeh Iran

**Keywords:** Aegyptianella, Haemoparasite, Haemoproteus, Leucocytozoon, Plasmodium

## Abstract

**Background:**

Avian haemosporidians (*Haemoproteus*, *Plasmodium*, and *Leucocytozoon*) and erythrocytic bacteria (e.g., *Aegyptianella*) are globally widespread, yet their epidemiology in Iran's semi‐arid ecosystems remains understudied.

**Objective:**

This study provides the first survey of avian blood parasites in Semnan Province, Iran, integrating microscopic and spatial analyses to identify host and environmental factors influencing infection patterns.

**Methods:**

From September 2022 to December 2023, 263 healthy birds from ten species were examined. Giemsa‐stained blood smears were screened microscopically. Infection hotspots and risk factors were identified using Kruskal–Wallis tests, logistic regression, and spatial mapping.

**Results:**

Overall infection prevalence was 22.1% and was dominated by *Haemoproteus* spp. (18.6%), followed by *Plasmodium* spp. (1.5%), *Leucocytozoon* spp. (1.1%), and *Aegyptianella* spp. (0.8%). Pigeons exhibited the highest infection rate and were nearly three times more likely to be infected than other species (OR = 2.78, 95% CI: 1.38–5.60, *p* = 0.004). Spatial analysis revealed infection clustering in Shahroud County, which exhibits relatively higher humidity and vegetation cover compared with other parts of the predominantly semi‑arid Semnan Province. These findings highlight the potential influence of local climatic and ecological factors on the distribution of avian haemosporidian parasites. Although infection appeared slightly higher during spring and summer (9.6% and 7.3%, respectively) compared with autumn (3.4%) and winter (1.9%), the observed differences were not statistically significant (*p* > 0.05).

**Conclusions:**

This baseline study provides important epidemiological data and establishes a framework for future molecular and vector‑based investigations of avian blood parasites in the Middle East.

## Graphical abstract

A survey of 263 birds across 10 species in Semnan Province, Iran, revealed a 22.1% prevalence of avian haemosporidian infections, dominated by *Haemoproteus* spp. Pigeons showed significantly higher infection risk. Spatial analysis identified clustering in Shahroud County, suggesting local climatic and ecological conditions influence parasite distribution in this semi‑arid region

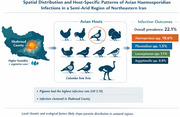

## 1 Introduction

Avian populations, both domestic and wild, are hosts to a wide variety of viral, bacterial, fungal, and parasitic pathogens that can substantially affect their health, productivity, and survival (Kobuszewska and Wysok 2024; Shan et al. 2022; Swangneat et al. 2025). Among parasitic agents, haemosporidian protozoa are particularly important due to their widespread occurrence and ecological impact. These blood parasites, belonging to the genera *Haemoproteus*, *Plasmodium*, and *Leucocytozoon*, infect avian erythrocytes and are transmitted by hematophagous vectors such as mosquitoes, blackflies, biting midges, and louse flies. In contrast, *Aegyptianella* spp. are intraerythrocytic rickettsial bacteria, primarily transmitted by ticks, and are taxonomically distinct from haemosporidian protozoa (Elahi et al. 2014; Köchling et al. 2023; Murdock et al. 2015).

More than 250 haemosporidian species have been described globally, exhibiting diverse transmission patterns and host specificities across ecological zones (Harl et al. 2020; Valkiūnas and Iezhova 2018, 2022, 2023). Although infections are often subclinical, they can cause anaemia, reduced fitness, impaired reproduction, and occasionally mortality, thereby influencing avian population dynamics and biodiversity conservation (Cruz et al. 2024; Schoenle et al. 2017). The severity and prevalence of infection are influenced by host factors (species, age, immunity) and environmental variables (season, altitude, and humidity), and the specific parasite species involved (Illera et al. 2017; Rodríguez‐Hernández et al. 2021; Vinagre‐Izquierdo et al. 2022). Climatic conditions strongly determine vector survival and parasite distribution, with infections reported from nearly all continents except Antarctica (Masello et al. 2018). In Iran, reported prevalence rates of avian haemosporidians range from 2% to 50%, with higher infection levels observed in the humid northern provinces (Nourani et al. 2018; Nourani et al. 2020) and lower rates in the arid central regions (Mirzaei et al. 2020). In the southern provinces, studies have reported prevalence rates of avian haemosporidians of 23.9%, 51.1%, and 55.7%, indicating significant levels of infection that warrant further investigation (Ghaemitalab et al. 2025; Ghaemitalab et al. 2021; Mohaghegh et al. 2018). However, data from northeastern Iran, a transitional zone between semi‐arid plains and mountainous ecosystems, remains scarce. Semnan province represents an ecologically heterogeneous region where climatic and environmental conditions may influence the distribution and transmission patterns of avian blood parasites. Despite this potential importance, no comprehensive epidemiological or spatial studies have investigated avian haemoparasites in this region, and the host range, prevalence, and spatial distribution of these parasites remain largely unknown (Ghaemitalab et al. 2021; Nourani et al. 2018).

Therefore, the present study was designed to address this knowledge gap by conducting the first epidemiological and spatial assessment of avian blood parasites in Semnan Province, northeastern Iran. Specifically, this study aimed to (i) determine the prevalence and diversity of avian haemoparasites among different bird species, (ii) evaluate host‐related factors associated with infection, and (iii) identify spatial patterns of infection using GIS‑based spatial analysis. Understanding these patterns can provide baseline epidemiological data for future molecular and vector‑based studies and improve our understanding of avian haemoparasite ecology in semi‑arid ecosystems.

## 2 Materials and Methods

### 2.1 Study Area

The study was conducted in eight counties across Semnan Province, northeastern Iran, covering more than 100 villages. A total of 263 native birds were sampled, including hens, roosters, pigeons, turkeys, ducks, quails, sparrows, *Columba livia livia*, magpies, and ostriches from 133 households on 69 farms. Semnan lies on the edge of the central desert and the Alborz mountain range, with elevations ranging from 1000 to 3000 m above sea level. The province covers 97 491 km^2^ and has a population of over 700 000. The climate varies from mild and cold in the north to hot and dry in the south, with generally low to moderate annual rainfall (http://amar.sci.org.ir).

### 2.2 Sample Collection

Between September 2022 and December 2023, a total of 263 clinically healthy birds representing ten species were sampled across various localities in Semnan Province using a convenience sampling approach, based on the accessibility of farms and the availability of birds during field visits. Blood samples were collected from the brachial vein using sterile 26‐gauge needles after disinfecting the puncture site with 70% ethanol. Only birds that appeared clinically healthy and had no history of antiparasitic treatment were included in the study to ensure that the observed prevalence reflected natural infection patterns and was not underestimated due to recent therapeutic interventions. Birds younger than 5 months were excluded to ensure that all sampled individuals had been exposed to at least one full transmission season, as very young birds may not have had sufficient time to develop detectable parasitaemia following vector exposure (Valkiunas 2005). Additionally, birds whose owners declined participation were excluded to comply with ethical standards and institutional animal welfare guidelines. Written informed consent was obtained from all owners before sampling. Free‐ranging sparrows, *C. livia livia*, and magpies were live‐trapped using baited cage traps for blood collection. Following sampling, all birds were safely released at the site of capture. For each bird, a data sheet was completed recording age, sex, species, geographic location, and sampling season.

### 2.3 Laboratory Analysis

Blood was collected from the wing vein using sterile equipment. Thin blood smears were air‐dried, fixed in methanol, and stained with 20% Giemsa solution for 20 min (Elahi et al. 2014; Mohaghegh et al. 2018). Slides were examined under 400× and 1000× oil immersion magnifications, where at least 100 microscopic fields per slide were evaluated for the presence of haemoparasites by two independent parasitologists. Parasitemia was estimated by counting the number of infected erythrocytes observed across 100 microscopic fields under oil immersion (1000×). The value was recorded as the number of infected cells per 100 examined fields, providing a relative estimate of parasite burden within each smear rather than a standardized erythrocyte‑based parasitaemia index (Acherar et al. 2024). Identification followed Valkiūnas’ taxonomic keys (Valkiunas 2005).

### 2.4 Statistical and Spatial Analysis

Data quality control procedures were performed before statistical analysis. Field data sheets and laboratory records were independently reviewed by two researchers to ensure consistency between original records and digital datasets. The dataset was screened for missing values, duplicate entries, and inconsistencies in demographic and sampling variables, including species, age, sex, season, and sampling location. Geographic coordinates recorded during field sampling were verified and standardized before import into ArcGIS. Any incomplete or inconsistent records were corrected using the original field forms or excluded when verification was not possible. Statistical analyses were performed using SPSS version 26.0 (IBM Corp., Armonk, NY, USA). The prevalence of infection was expressed as frequency, percentage, and 95% confidence intervals (CIs). Differences in parasitaemia levels among bird species were evaluated using the Kruskal–Wallis test, followed by appropriate multiple comparison tests where applicable. To assess the association between infection status and potential risk factors, including age, sex, season, and location, binary logistic regression models were applied. All selected variables were included simultaneously in the multivariable model. Interaction terms were not examined because of the limited sample size in several species categories and the large number of potential comparisons, which could compromise model stability and result in unreliable estimates. Model adequacy was assessed using basic diagnostic procedures, including evaluation of multicollinearity and overall goodness of fit, which indicated an acceptable fit of the model to the data. Statistical significance was defined at *p* < 0.05. Spatial visualization and spatial autocorrelation analysis of parasite distribution were conducted using ArcGIS Desktop version 10.8.2 (ESRI, Redlands, CA, USA). Laboratory data containing county name, parasite species, and number of positive cases were imported into ArcGIS in Microsoft Excel format.

For spatial visualization, counties were colour‐coded according to infection intensity (based on the percentage of positive samples) to display the overall geographic distribution of infections across Semnan Province. Moreover, to formally assess spatial autocorrelation, global Moran's *I* was calculated using the Spatial Statistics toolbox in ArcGIS. The analysis was performed on the coordinates of individual birds, using a binary infection status variable (0 = negative, 1 = positive). A fixed distance band conceptualization was applied, and statistical significance was evaluated using 999 permutations. In general, a Moran's *I* value close to +1.0 indicates a clustered distribution, a value near 0 suggests a random spatial distribution, and a value approaching −1.0 indicates a dispersed (or regular) pattern (Gholizadeh et al. 2017).

## 3 Result

A total of 263 birds representing several species from different localities of Semnan Province, Iran, were examined for avian haemoparasites.

Microscopic examination revealed infections with three genera of haemosporidian parasites (*Haemoproteus*, *Plasmodium*, and *Leucocytozoon*) as well as erythrocytic bacteria of the genus *Aegyptianella* (Table 1; Figure 1). The overall prevalence of haemosporidian infections was 22.1%, with *Haemoproteus* being the dominant parasite (18.6%), followed by *Plasmodium* (1.5%) and *Leucocytozoon* (1.1%). In addition, *Aegyptianella* spp. were detected in 0.8% of examined birds.

**TABLE 1:** Prevalence of avian haemosporidian infections and *Aegyptianella* spp. in birds from Semnan Province by sex, age, season, location, and species.

Factor		No. examined	*Haemoproteus*	*Plasmodium*	*Leucocytozoon*	*Aegyptianella*
Sex	Female	164 (62.4%)	31 (11.8%)	4 (1.5%)	1 (0.4%)	1 (0.4%)
	Male	99 (37.6%)	18 (6.8%)	—	2 (0.8%)	1 (0.4%)
Age	Young	166 (63.1%)	32 (12.2%)	3 (1.1%)	2 (0.8%)	1 (0.4%)
	Adult	97 (36.9%)	17 (6.5%)	1 (0.4%)	1 (0.4%)	1 (0.4%)
Season	Spring	107 (40.7%)	20 (7.6%)	2 (0.8%)	1 (0.4%)	2 (0.8%)
	Summer	78 (29.7%)	16 (6.1%)	1 (0.4%)	2 (0.8%)	—
	Autumn	47 (17.9%)	8 (3.0%)	1 (0.4%)	—	—
	Winter	31 (11.8%)	5 (1.9%)	—	—	—
Location	Shahroud	102 (38.8%)	18 (6.8%)	1 (0.4%)	—	1 (0.4%)
	Garmsar	59 (22.4%)	10 (3.8)	2 (0.8%)	1 (0.4%)	1 (0.4%)
	Semnan	39 (14.8%)	9 (3.4%)	—	—	—
	Mayamey	33 (12.5%)	6 (2.3%)	1 (0.4%)	1 (0.4%)	—
	Damghan	30 (11.4%)	6 (2.3%)	—	1 (0.4%)	—
Bird	Pigeon	52 (19.8%)	26 (9.9%)	—	1 (0.4%)	—
	Hen	51 (19.4%)	11 (4.2%)	—	1 (0.4%)	—
	Quail	31 (11.8%)	3 (1.1%)	1 (0.4%)	1 (0.4%)	1 (0.4%)
	Ostrich	28 (10.6%)	3 (1.1%)	1 (0.4%)	—	—
	Rooster	17 (6.5%)	2 (0.8%)	—	—	—
	Magpie	11 (4.2%)	2 (0.8%)	1 (0.4%)	—	—
	Duck	4 (1.5%)	—	1 (25.0%)	—	1 (50.0%)
	Sparrow	4 (1.5%)	1 (0.4%)	—	—	—
	Turkey	4 (1.5%)	—	—	—	—
	*Columba livia livia*	3 (1.1%)	1 (0.4%)	—	—	—

**FIGURE 1:**
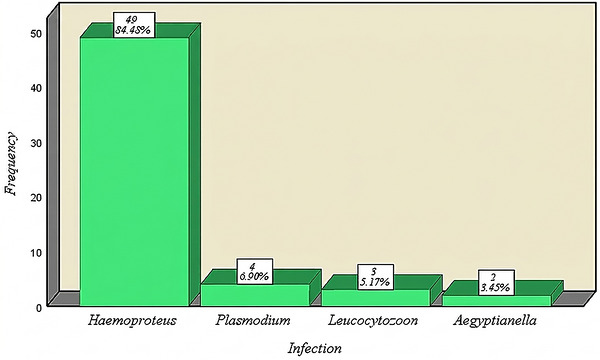
Prevalence of different haemoparasites and *Aegyptianella* spp. in birds from Semnan Province, Iran.

### 3.1 Distribution by Demographic and Ecological Variables

As shown in Table 1 and Figure 2A, infection rates were slightly higher in females (14.1%) than in males (8.0%), though this difference was not statistically significant (OR = 1.03, 95% CI: 0.53–2.01, *p* = 0.94; Table 2). Similarly, young birds showed a somewhat higher infection rate (14.4%) than adults (7.6%), but the difference was not significant (OR = 1.11, 95% CI: 0.58–2.12, *p* = 0.76). Seasonal differences in infection prevalence were not statistically significant, although the highest prevalence was observed in spring (9.5%) and summer (7.2%), whereas the lowest prevalence occurred in winter (1.9%) (Figure 2B).

**FIGURE 2:**
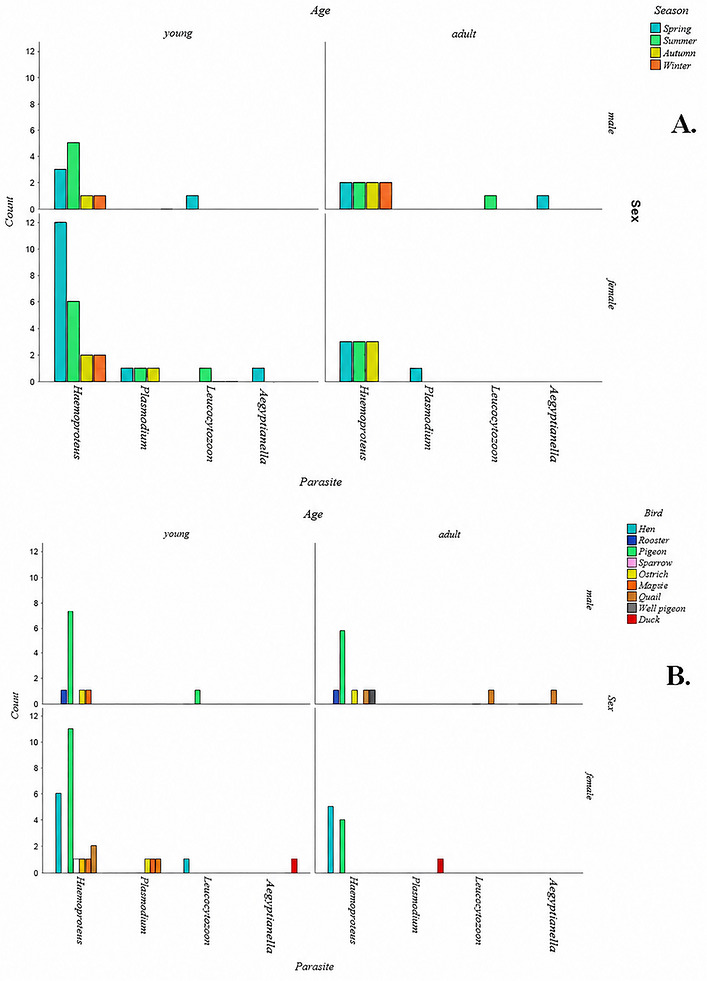
(A) Comparison of parasite infection rates by age, sex, and season in infected birds. (B) Comparison of parasite infection rates by age, sex, and species in infected birds.

**TABLE 2:** Odds of avian haemosporidian infections and *Aegyptianella* spp. according to demographic variables in birds from Semnan Province, Iran.

Factor	Odds ratio	95% CI for odds ratio	*p*‐value
Sex (ref = male)			
Female	1.03	(0.53–2.01)	0.941
Age (ref = adult)			
Young	1.11	(0.58–2.12)	0.764
Location (ref = Shahroud)			
Garmsar	0.87	(0.33–2.33)	0.781
Semnan	0.81	(0.28–2.38)	0.701
Mayamey	0.84	(0.37–1.90)	0.667
Damghan	0.94	(0.33–2.65)	0.898
Bird (ref = others)			
Pigeon	**2.78**	**(1.38–5.60)**	**0.004**
Hen	1.23	(0.52–2.88)	0.644
Season (ref = winter)			
Spring	1.71	(0.57–5.14)	0.343
Summer	1.64	(0.53–5.07)	0.389
Autumn	1.22	(0.35–4.20)	0.757

### 3.2 Species‐specific Infection Patterns

Among all examined species (Table 1), pigeons exhibited the highest infection rate (10.3%), followed by hens (4.6%) and quails (2.3%). Representative micrographs of *Haemoproteus* gametocytes detected in domestic hens are shown in Figure 3. Statistical analysis revealed a significant association between bird species and infection prevalence (OR = 2.78, 95% CI: 1.38–5.60, *p* = 0.004; Table 2).

**FIGURE 3:**
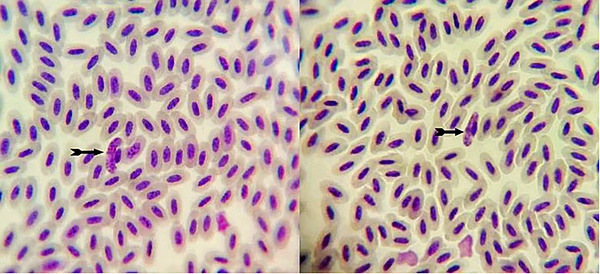
Light micrograph of a *Haemoproteus* gametocyte observed in a Giemsa‐stained blood smear from a domestic hen under oil immersion (1000×). The parasite is indicated by arrows.

### 3.3 Spatial and Geographical Distribution

GIS‑based spatial visualization demonstrated heterogeneity in infection patterns across the province (Figures 4, 5, 6, 7). The highest infection intensities were observed in Shahroud and Garmsar counties (Figure 7), whereas Sorkheh, Aradan, and Mahdishahr showed minimal or no infections. Colour‐coded mapping (Figure 5) indicated clear positive cases in the northern and eastern parts of Semnan Province, suggesting that local ecological and climatic factors may promote parasite transmission. To formally assess spatial clustering, global Moran's *I* was calculated using the GPS coordinates of all sampled birds. The analysis yielded a Moran's *I* of −0.123 (*p* = 0.134), indicating no statistically significant spatial autocorrelation in infection status across the study region.

**FIGURE 4:**
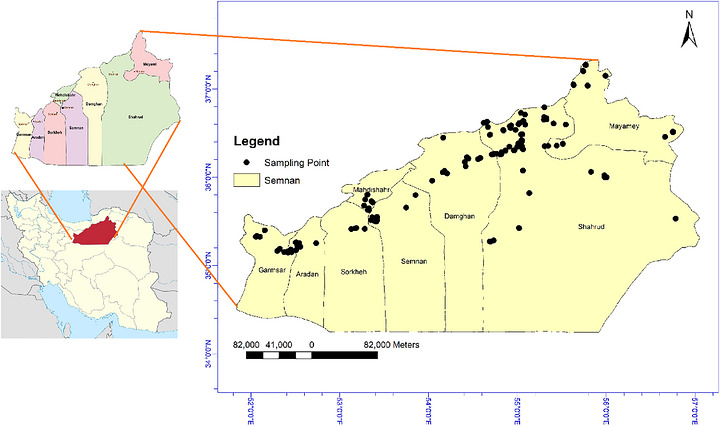
Spatial distribution of bird blood sampling points in Semnan Province, Iran.

**FIGURE 5:**
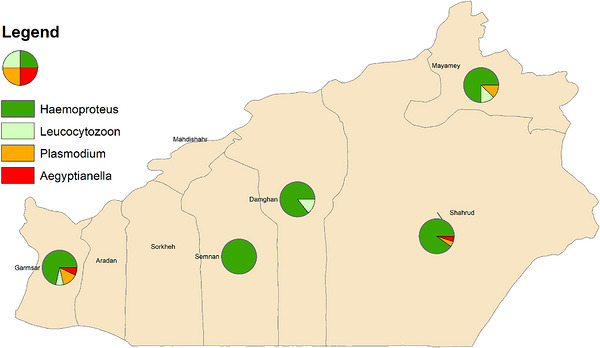
Spatial distribution of different species of avian haemoparasites and *Aegyptianella* spp. in the counties of Semnan Province, Iran.

**FIGURE 6:**
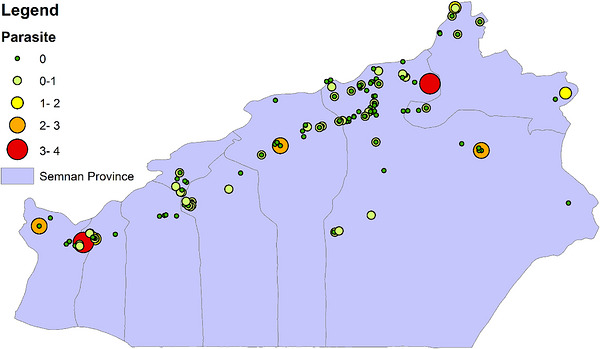
Spatial distribution and intensity of avian haemoparasite and *Aegyptianella* spp. infections in Semnan Province, Iran.

**FIGURE 7:**
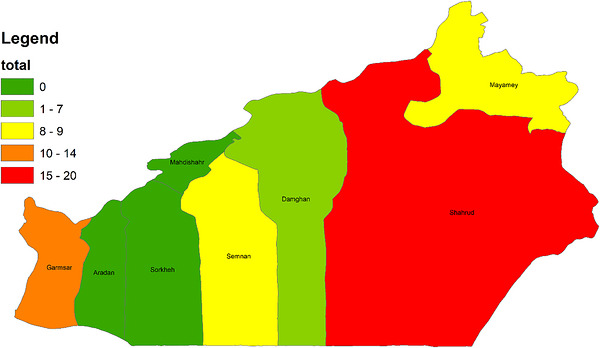
Spatial distribution of avian haemoparasite and *Aegyptianella* spp. infection levels across the counties of Semnan Province, Iran.

### 3.4 Parasitemia Levels and Parasite Composition

As shown in Figure 8, mean parasitaemia levels differed significantly among bird species (*p* < 0.05), with pigeons and hens showing the highest parasite loads. The genus *Haemoproteus* predominated across all sampling sites (Figure 5), whereas *Plasmodium* and *Leucocytozoon* occurred sporadically in warmer lowland regions.

**FIGURE 8:**
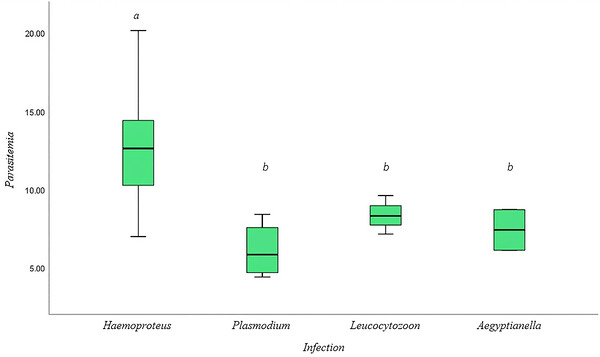
Mean parasitaemia levels of haemoparasitic infections and *Aegyptianella* spp. in different bird species. Significant differences between species are indicated by distinct letters (*p* < 0.05).

## 4 Discussion

This study represents the first survey of avian haemosporidian parasites in Semnan Province, northeastern Iran, revealing a relatively low to moderate prevalence (22.1%). The dominance of *Haemoproteus* spp. (18.6%) and the limited occurrence of *Plasmodium* and *Leucocytozoon* spp. is consistent with a possible influence of the semi‐arid ecological and climatic characteristics of this region. Such conditions could hypothetically favour transmission of *Haemoproteus* by biting midges (*Culicoides*) and louse flies (Hippoboscidae), while being less suitable for mosquito and blackfly vectors. However, given the absence of direct measurements of environmental variables (temperature, precipitation, vegetation cover) and vector abundance, this interpretation remains speculative and should be confirmed by further studies. In addition, *Aegyptianella* spp., detected at a low prevalence (0.8%), are intraerythrocytic, tick‑associated bacteria of birds. Available evidence indicates that transmission is primarily linked to ticks, with reports most commonly implicating soft ticks (*Argas* spp.) and, in some avian settings, hard ticks such as *Hyalomma* spp. Consequently, the occurrence of *Aegyptianella* is expected to track tick abundance and host–tick contact patterns rather than the dipteran vectors (Culicoides, hippoboscid flies, mosquitoes, and blackflies) that drive haemosporidian transmission (Elahi et al. 2014; Köchling et al. 2023; Murdock et al. 2015).

The infection pattern observed here aligns with previous studies in Iran, which reported higher prevalences in humid northern and southern provinces (23%–51%) and lower values in central arid regions (10%–12%) (Fakhar et al. 2013; Ghaemitalab et al. 2021; Mirzaei et al. 2020; Mohaghegh et al. 2018; Nourani et al. 2018). This trend is consistent with the well‑established association between environmental humidity, vector availability, and haemosporidian transmission intensity. The higher infection rates detected in Shahroud County, an area characterized by relatively greater vegetation and cooler microclimates, may further reflect the influence of local ecological conditions on parasite distribution (Clark et al. 2020; Fecchio et al. 2021; Vinagre‐Izquierdo et al. 2022).

Among the examined species, pigeons exhibited the highest infection prevalence (10.3%) and were nearly three times more likely to be infected compared with other bird species (OR = 2.78, *p* = 0.004). This finding is consistent with reports identifying *Haemoproteus columbae* as a highly prevalent parasite in pigeons globally (Alkharigy et al. 2018; Nourani et al. 2021; Quillfeldt et al. 2011). The elevated parasitaemia levels observed in pigeons and hens may suggest a potential role in maintaining parasite circulation within mixed avian populations. However, since our study was limited to measuring infection prevalence without investigating parasite persistence, transmission dynamics, or vector competence, the reservoir status of these species cannot be confirmed (Chaechi‐ Nosrati et al. 2018; Refaat et al. 2020).

Turkeys and ducks in our study were free of *Haemoproteus* spp., whereas quails were the only birds infected with all four parasite types. Because all birds experienced similar climatic conditions, these differences likely reflect species‐specific susceptibility rather than weather effects (Strehmann et al. 2023). The prevalence of haemosporidian parasites worldwide depends on multiple factors, including vector presence, sampling season, host immunity, genetics, nutrition, and climatic variables such as altitude, humidity, temperature, and rainfall (Bensch et al. 2012; Garcia‐Longoria et al. 2019; Rivero de Aguilar et al. 2018).

The prevalence of *Plasmodium* spp. (1.5%) and *Leucocytozoon* spp. (1.1%) was markedly lower than that reported in more humid regions, which may be consistent with the hypothesis that mosquito and blackfly vectors are limited under the climatic conditions of Semnan Province. Similarly, the low frequency of *Aegyptianella* spp. infections (0.8%) may align with reports from other semi‐arid regions, where tick abundance tends to be lower (Rahmani et al. 2022; Short et al. 2017).

Although infection rates were slightly higher in females and young birds, these differences were not statistically significant. This pattern may reflect transient susceptibility during physiological stress (breeding) or incomplete immune development rather than strong demographic effects (Lachish et al. 2011). Seasonal variation also followed expected trends, with higher infection prevalence during spring and summer periods, which could correspond to increased vector activity but without statistical significance, likely due to sample size limitations (Cheke et al. 1976; Gupta et al. 2011).

Overall, the spatial and host‐specific heterogeneity observed in this study emphasizes the multifactorial nature of avian haemosporidian epidemiology. However, the spatial analyses performed in this study were exploratory in nature and were intended primarily to describe geographic patterns of infection rather than to identify causal environmental predictors or develop spatial risk models. Incorporating molecular detection and vector monitoring in future studies will help clarify cryptic infections and reveal potential transmission cycles specific to northeastern Iran's semi‐arid ecosystems. Although higher infection prevalence was observed in Shahroud County, formal spatial autocorrelation analysis did not detect statistically significant clustering (Moran's *I* = −0.123, *p* = 0.134). This may reflect the relatively low number of positive cases (*n* = 58), uneven sampling effort across counties, or a true absence of spatial structure in parasite transmission in this semi‐arid ecosystem.

The present study relied exclusively on microscopic examination of Giemsa‑stained blood smears. Although microscopy remains a widely used diagnostic method in avian parasitology, it has lower sensitivity compared with molecular techniques such as PCR. Consequently, infections with low parasitaemia or chronic infections may have been underestimated. In addition, microscopic examination often limits parasite identification to the genus level, making precise species‐level identification difficult and potentially overlooking mixed or cryptic infections. Future studies incorporating molecular detection methods and vector monitoring would provide more accurate parasite identification and help clarify transmission dynamics in the semi‑arid ecosystems of northeastern Iran.

In addition, parasitaemia was estimated based on the number of infected erythrocytes observed across a fixed number of microscopic fields rather than the conventional method of counting infected cells per 10,000 erythrocytes. Because erythrocyte density can vary among smears depending on preparation quality and smear thickness, this approach may introduce variability in parasitaemia estimates and limit direct quantitative comparisons with studies that apply standardized erythrocyte‑based counting methods. Another important limitation of this study is the uneven sampling effort across bird species. Because sample sizes were determined by local availability, some species (such as pigeons and domestic hens) were represented by relatively large numbers, while others (including ducks, sparrows, and turkeys) had very small sample sizes. Consequently, species‑specific prevalence values should be interpreted cautiously, as estimates for species with limited sample sizes may not reliably reflect true infection rates and are not suitable for robust statistical comparisons.

## 5 Conclusion

This study provides the first baseline data on the diversity and prevalence of avian blood parasites in Semnan Province, Iran. The dominance of *Haemoproteus* spp. and the observed geographic variation in infection prevalence across ecologically favourable northern areas suggest a possible role of environmental and host‐related factors in shaping transmission dynamics. The findings suggest that pigeons and hens, which exhibited comparatively high prevalence and parasitaemia, could potentially contribute to sustaining transmission of certain haemosporidian lineages in the local avian community. Future research integrating molecular diagnostics, vector ecology, and climatic modelling is essential to elucidate transmission pathways and assess the potential ecological and health impacts of haemosporidian infections in domestic and wild birds of arid regions.

## Author Contributions

**Seyed‐Reza Mirbadie**: Software, conceptualization, data curation, formal analysis, investigation, validation, visualization, writing – original draft. **Rasoul Alimi**: Formal analysis, software, visualization, writing – original draft. **Eissa Soleymani**: Methodology, visualization, writing – original draft. **Naghmeh Dastan**: Data curation, writing – original draft. **Abdolmajid Gholizadeh**: Formal analysis, software, writing – original draft. **Amir‐Hossein Maghsood**: Conceptualization, data curation, formal analysis, investigation, methodology, project administration, validation, visualization, writing – original draft, writing – review and editing. **Mohammad‐Ali Mohaghegh**: Conceptualization, formal analysis, investigation, methodology, validation, visualization, writing – original draft, writing – review and editing.

## Funding

This study was financially supported by Hamadan University of Medical Sciences (Project No. 1401011672).

## Ethics Statement

The study was approved by the Ethical Review Committee of Hamedan University of Medical Sciences (IR.UMSHA.REC.1400.953). Written informed consent was obtained from animal owners.

## Conflicts of Interest

The authors declare no conflicts of interest.

## Data Availability

The data that support the findings of this study are available from the corresponding author upon reasonable request.
